# How gacha gaming and life quality shape problem gambling risk: insights from a cross-sectional study using Hong Kong-based online survey of young adults

**DOI:** 10.3389/fpsyt.2025.1663328

**Published:** 2025-09-22

**Authors:** Anson Chui Yan Tang, Rick Yiu Cho Kwan, Eliza Mi Ling Wong, Winnie Lai Sheung Cheng

**Affiliations:** ^1^ School of Nursing, Tung Wah College, Hong Kong, Hong Kong SAR, China; ^2^ S.K.Yee School of Health Sciences, Saint Francis University, Tseung Kwan O, Hong Kong SAR, China

**Keywords:** gacha gaming, quality of life, problem gambling, Chinese, young adults

## Abstract

**Background:**

Research related to the effects of gacha gaming on problem gambling among Chinese young adults in Hong Kong is limited the impact of quality of life (QoL) domains on gacha-related gambling behaviors remains largely unexplored. This study explored associations between gacha gaming behaviors QoL problem gambling risk in this population.

**Methods:**

**A** cross-sectional study used an online survey to collect data from 281 young adults (aged 18–25) with experience in freemium gaming. Participants completed questionnaires on socio-demographics, problem gambling risk (PGSI-C), QoL (WHOQOL-BREF, Hong Kong version), and gacha gaming behaviors (e.g., daily gaming time, monthly expenses). Stepwise regression analyzed associations between PGSI scores, QoL domains, and gaming behaviors, with p<0.05 indicating significance.

**Results:**

Of 281 respondents, 63.3% belonged to the low-risk problem gambling group, and 11% belonged to the high-risk group. High-risk gamers had significantly higher monthly gacha expenses (p=0.021). Regression analysis revealed a significant association between PGSI scores, daily gaming time, QoL variables, and education level(Adjusted R²=0.113, p=0.001). Physical and overall QoL were negatively associated with problem gambling risk(p<0.01), while daily gaming time and social QoL were positively associated with problem gambling risk(p<0.05,p<0.01). Effect sizes of all significant variables were small (f²=0.014–0.04).

**Conclusions:**

The positive association between social QoL and problem gambling risk suggests that a gamer’s social circle significantly influences gambling behavior. These findings provide direction for future studies on the contributing roles of different QoL domains in gacha-related gambling among Chinese young adults in Hong Kong. Future studies shall adopt a probability sampling approach and/or a wider sampling pool to increase the generalizability of the findings.

## 1 Introduction

Gacha games are a prominent component of the freemium mobile gaming sector in Asia. Their widespread popularity has drawn significant concern from healthcare professionals due to potential adverse effects on the mental wellbeing of young adults (1, 2). Originating from Japanese “gachapon” toy vending machines, the gacha model has become a defining feature of contemporary mobile gaming. The global gacha games market was valued at US$452 million in 2023, with projections suggesting growth to US$781.5 million by 2030 (3). China is a major contributor to this market, generating over US$21 billion in mobile gacha revenue in 2020 alone (4). In Hong Kong, the accessibility of gacha games on smartphones raises concerns about compulsive spending, which corresponds to problem gambling. It is therefore essential to investigate gacha gaming in Hong Kong and its association with gambling tendencies to guide preventive strategies for identifying and supporting at-risk individuals.

Gacha games are similar to loot boxes, which are prevalent in Western gaming markets, as both employ randomized reward mechanisms (RRMs) (e.g. pulling, rolling) that can trigger gambling behaviors (5, 6). While loot boxes are typically found in console or PC games, gacha games are predominantly played on smartphones, making them more accessible and reducing the initial entry cost. To progress in gacha games, gamers spend virtual currencies to obtain random items, such as rare characters or powerful equipment (1, 7). Games like *Genshin Impact* utilize psychological tactics, including rarity tiers and limited-time events, to heighten anticipation, thereby encouraging spending patterns that resemble gambling (8, 9). This “pay-to-win” model promotes real-money transactions, transforming the gaming experience into a gambling activity that can lead to uncontrollable spending and result in problem gambling (9, 10). Furthermore, this model exploits psychological vulnerabilities, such as the illusion of control and fear of missing out (FOMO), to drive in-game spending (11, 12). These tactics have been criticized for encouraging continuous expenditure, which escalates the risk of diminished self-control, financial harm, and risk of problem gambling (2, 13).

Extant research demonstrates a significant link between spending on loot boxes and risk of problem gambling behaviors. Studies consistently find that individuals who spend more on in-game purchases tend to score higher on problem gambling (14, 15). For instance, Wardle and Zendle (2020) reported that purchasing loot boxes is significantly associated with a greater risk of problem gambling among British young adults aged 16–24 (16). Longitudinal studies also suggest that engagement with loot boxes may predict future online gambling activities (17). Additionally, research indicates connections between loot box purchases and increased levels of Gaming Disorder, anxiety, and depression (8). Some studies show that a preoccupation with randomized rewards encourages gambling behavior, reduces self-control, and increases depressive symptoms (11, 18). A systematic review by Raneri et al. (2022) concluded that loot boxes pose greater problem gambling risks than other forms of microtransactions. It highlights the need to examine gacha games due to their similar mechanics with loot boxes (19). However, research on gacha games remains scarce. In Hong Kong, only one cross-sectional study has been identified, in which Tang et al. (2022) found that 25% of young adult gacha gamers were found to have high problem gambling risk (20).

The study by Tang et al. (2022) (20) also revealed that stress and anxiety are significantly associated with problem gambling risk. Similarly, research on gaming has documented negative associations between Gaming Disorder and psychological health indicators such as anxiety, stress, and depression (21). Gaming Disorder has also been linked to more severe mental and social issues, including suicidal ideation, increased social anxiety, and poorer school integration (22, 23). This evidence suggests that the effects of Gaming Disorder extend beyond mental health to impact overall quality of life (QoL). The World Health Organization (WHO) defines QoL as an individual’s perception of their life circumstances within their cultural and value context, relative to their goals, expectations, and concerns, encompassing physical health, psychological state, social relationships, and environmental factors (24). The systematic review conducted by Noroozi et al. (2021) (25) confirms the significant negative associations between Gaming Disorder and QoL across various domains. Likewise, Jeong et al. (2021) found that excessive gaming impairs daily functioning and reduces QoL among Korean adults (26). In a Hong Kong study, Kwok et al. (2021) identified a negative association between Gaming Disorder, psychological QoL, and academic performance among university students (27). In gambling research, Loo et al. (2016) found that problem gamblers had significantly lower scores in all WHO-defined QoL domains as compared to non-problem gamblers in young adult populations in Macau and Australia (28). Some studies suggest a bidirectional relationship between QoL and risk behaviors. For instance, de Oliveira Pinheiro et al. (2020) (29) and Ferrari Junior et al. (2024) (30) showed that QoL domains were negatively associated with risk behaviors such as problem gambling and Gaming Disorder. A systematic review by Paulus et al. (2018) (31) further supports that QoL may influence gaming and gambling behavior in adolescents. Paulus and colleagues (31) found that external factors, including familial (e.g., parental relationships and support) and social factors (e.g., peer interactions), significantly contribute to the development of Gaming Disorder. These external factors also shape an individual’s perceived QoL. These findings underscore the negative impact of Gaming Disorder and problem gambling on an individual’s quality of life, while also suggesting that pre-existing QoL may influence the development of these behaviors.

Currently, there is limited research focused on the effects of gacha gaming on problem gambling among Chinese young adults in Hong Kong. Furthermore, the impact of quality of life on gacha-related gambling behaviors remains largely unexplored. This lack of region-specific evidence underscores the urgent need for studies to investigate the interplay between QoL, gacha gaming and problem gambling.

## 2 Objective

The present study aimed to investigate the associations between gacha gaming behaviors, quality of life, and the risk of problem gambling among Chinese young adult gamers. We hypothesized that both gacha gaming behaviors and quality of life would be significantly associated with problem gambling risk in this population. The manuscript was written in accordance with STROBE guideline (32).

## 3 Significance

Exploring the interplay between gacha gaming, quality of life, and problem gambling risk is crucial for unravelling how these factors collectively influence gacha-related gambling behaviors. From a research perspective, this study aims to contribute to the existing body of knowledge by suggesting the potential value of incorporating QoL measures into the assessment of problem gambling risks associated with gacha gaming. Practically, the findings could offer valuable insights for healthcare professionals, including mental health nurses and primary care providers, regarding the influence of QoL on problem gambling risk in gacha gamers. This knowledge may, in turn, aid in refining screening tools for at-risk individuals in primary healthcare settings, such as youth centers and community gambling prevention centers in Hong Kong.

## 4 Materials and methods

### 4.1 Study design, setting and participants

This study employed a cross-sectional design, collecting data online using Google Forms. The target population comprised young adults aged 18 to 25 who reported playing freemium games at least once a week over the past 12 months. Convenience and snowball sampling were employed to recruit participants over six months through personal connections, two social media platforms (i.e., Facebook and Instagram), and two gaming discussion forums (i.e., LIHKG-Game Zone and Games Animation Forum). Individuals who could not read Chinese were excluded. To prevent missing data, all questions in the online survey were made mandatory, ensuring a complete dataset for analysis.

Before conducting the survey, participants were presented with a clear description of the study’s objectives. Informed consent was obtained online. To indicate their voluntary agreement, participants were required to select a checkbox labeled “Agree to participate in this study.” This action was a mandatory prerequisite for beginning the survey. Ethical approval for this study was granted by the ethics committee of the first author’s affiliated institution.

### 4.2 Study size

G*Power version 3.1.9.7 was used to calculate the sample size. Due to the limited prior evidence, conventional study power and effect size, as described by Cohen (1992) (33), were used to calculate the sample size. The minimum sample size required to achieve a study power of 0.8, with an alpha of 0.05 and a medium effect size for association (f²=0.15), was 131 for 13 independent variables (30). The 13 variables included four socio-demographic variables (i.e., age, gender, education level, and monthly income), four gaming-related variables (i.e. daily gaming time, number of gacha games played, frequency of gacha purchases, and monthly spending on gacha purchases), and five QoL-related variables (i.e. overall QoL and the four QoL domains). A total of 281 participants were recruited, which was more than sufficient for hypothesis testing.

### 4.3 Variables

#### 4.3.1 Risk of problem gambling

The Problem Gambling Severity Index – Chinese version (PGSI-C) was used to operationalize the risk of problem gambling. PGSI-C is a 9-item questionnaire that assesses an individual’s risk of problem gambling based on their gambling behavior and the associated adverse consequences (34). Each item is rated on a 4-point Likert scale, ranging from 0 (“never”) to 3 (“almost always”). The total PGSI-C score is calculated by summing all item ratings, resulting in a score range of 0 to 27. The total PGSI-C score can be categorized into three levels of problem gambling risk: 0–2 indicates a non/low-risk problem gambling; 3–7 signifies a moderate-risk problem gambling; and a score of 8 or higher represents a high-risk problem gambling. The PGSI-C demonstrated good concurrent, discriminant, and predictive validity, as well as good reliability, with a Cronbach’s alpha of 0.77 and a test-retest reliability (r) of 0.954 in the Chinese adult population (34).

#### 4.3.2 Quality of life

The 26-item Hong Kong Chinese World Health Organization Quality of Life instrument [WHOQOL-BREF (Hong Kong version)] was used to measure quality of life (35). This instrument is designed to assess quality of life across four domains: physical health, psychological health, social relationships, and the environment. It contains 24 items corresponding to these domains, along with two additional items that measure overall quality of life and general health. Each item is rated on a five-point Likert scale reflecting intensity, capacity, frequency, or evaluation. The total score for each domain is calculated according to the WHOQOL-BREF (HK) manual (35). The score range for overall QoL is 1-5, while the score ranges for the four QoL domains are 4-20. The instrument has demonstrated good reliability and validity. Test-retest reliability showed intraclass correlation coefficients ranging from 0.73 to 0.90 across domains, and its Cronbach’s alpha was ≥0.7 for all domains (36).

#### 4.3.3 Gacha gaming behaviors

Daily time spent on gacha gaming (i.e. ≤1hours, 1-3hours, 4-6hours, ≥6hours), the number of gacha games played (i.e. 1, 2-3, ≥4), the frequency of gacha purchase (i.e. never, once a day, once a week, once a month, once for half a year, once a year), and the monthly expenses on gacha purchase (i.e. ≤HKD100, HKD100-499, HKD500-999, ≥HKD1000) were designed to measure the participants’ gacha gaming pattern. Each item was measured on an ordinal scale.

#### 4.3.4 Socio-demographics

Age (i.e. 18–20 years/21–25 years), gender (i.e. male/female), and education level (i.e. secondary school, associate degree/higher diploma, bachelor’s degree or above) were collected as they were reported to be associated with the risk of problem gambling in video game microtransactions. Monthly income was also collected. Gender was measured in nominal while the rest were on an ordinal scale.

### 4.4 Statistical methods

SPSS version 29 was used to conduct all statistical analyses. There was no missing data in the dataset. Descriptive statistics were calculated to summarize all socio-demographic and other study variables among the problem gambling risk levels. Means and standard deviations were reported for continuous variables, while frequencies and percentages were computed for categorical and ordinal variables. To assess group differences, the Chi-squared test was used for gender, the Kruskal-Wallis test was applied to ordinal variables (i.e., education level, monthly income, and the four gaming behavior variables), and One-way ANOVA was utilized for overall quality of life and its four domain scores. *Post hoc* analysis with Bonferroni correction for multiple tests was performed to examine specific group differences for continuous variables. To verify the study’s hypothesis, Pearson Correlation Analysis or Spearman Rank Correlation Analysis was used, depending on the data’s normality, to explore correlations between education level, monthly income, gaming behaviors, quality of life and total problem gambling scores. Stepwise regression analysis was conducted with the total problem gambling score as the dependent variable to identify significant predictors. A two-sided p-value below 0.05 was considered statistically significant.

## 5 Results

### 5.1 Participants profile

A total of 281 participants were collected with no missing data. Of the participants, 43% (n=121) were male and 56.9% were female. The majority of the participants aged 21–25 years (57.7%, n=162) and held a bachelor’s degree or higher (78.6%, n=221). Regarding problem gambling risk, 63.3% (n=178) were classified as low-risk, 25.6% (n=72) as moderate-risk, and 11% (n=31) as high-risk.

As detailed in **Table 1**, significant differences were observed among the three problem gambling groups for age (χ²=12.657, p=0.002), gender (χ²=8.214, p=0.016), and education level (H=6.223, p=0.045). The high-risk group was composed of a greater proportion of older male participants as compared to the low- and moderate-risk groups. Among the gacha gaming behaviors, only monthly gacha expenses differed significantly across the groups (H=7.688, p=0.021). It was also noted that 38.7% of the high-risk group had a monthly income of HKD 10,000 or more. 87.5–89.3% of those in the low- and moderate-risk groups reported incomes of HKD10,000 or below.

**Table 1:** Comparison among the three risk levels of problem gambling for socio-demographic, gaming and quality of life variables (N=281).

Study variables	Low-risk problem gambler (n=178)	Moderate-risk problem gambler (n=72)	High-risk problem gambler (n=31)	*P* value
Age^a^, n(%)				
18–20 years	80(44.9)	35(48.6)	4(12.9)	0.002*
21–25 years	98(55.1)	37(51.4)	27(87.1)	
Gender^a^, n (%)				
Male	66(37.1)	36(50)	19(61.3)	0.016*
Female	112(62.9)	36(50)	12(38.7)	
Education level^c^, n (%)				
Secondary school	9(5.1)	13(18.1)	2(6.5)	0.045*
Associate Degree/Higher Diploma	23(12.9)	9(12.5)	4(12.9)	
Bachelor’s degree or above	146(82)	50(69.4)	25(80.6)	
Monthly income^c^, n(%)				
≤ HKD 10,000	159(89.3)	63(87.5)	19(61.3)	0.001*
HKD 10,000 to 19,999	14(7.9)	7(9.7)	11(35.5)	
HKD 20,000 to 39,999	5(2.8)	1(1.4)	1(3.2)	
HKD 40,000 or above	0(0)	1(1.4)	0(0)	
Daily time spent on gacha games^c^, n (%)				
≤ 1hour	42(23.6)	20(27.8)	2(6.5)	0.068
1–3 hours	116(65.2)	42(58.3)	23(74.2)	
4–6 hours	15(8.4)	8(11.1)	5(16.1)	
≥ 6 hours	5(2.8)	2(2.8)	1(3.2)	
Number of gacha games played^c^, n (%)				
1	81(45.5)	33(45.8)	9(29)	0.402
2-3	79(44.4)	36(50)	21(67.7)	
≥ 4	18(10.1)	3(4.2)	1(3.2)	
Frequency of gacha purchases^c^, n (%)				
Never	23(12.9)	6(8.3)	0(0)	0.08
Once a day	3(1.7)	0(0)	3(9.7)	
Once a week	13(7.3)	3(4.2)	2(6.5)	
Once a month	76(42.7)	30(41.7)	18(58.1)	
Once for half a year	44(24.7)	20(27.8)	7(22.6)	
Once a year	19(10.7)	13(18.1)	1(3.2)	
Monthly expenses on gacha purchase^c^, n (%)				
≤ HKD 100	85(47.8)	31(43.1)	8(25.8)	0.021*
HKD 100-499	64(36)	34(47.2)	12(38.7)	
HKD 500-999	15(8.4)	6(8.3)	8(25.8)	
HKD 1000 or above	14(7.9)	1(1.4)	3(9.7)	
PSGI score^b^, M(SD)	0.37(0.64)	4.32(1.3)	10.87(3.64)	0.001*
WHOQOL-BREF(HK) domain scores^b^, M(SD)				
QoL(overall)	3.40(0.77)	3.25(0.82)	2.61(0.95)	0.001*
QoL(Physical)	14.88(2.01)	14.76(2.11)	13.13(1.61)	0.001*
QoL(Psychological)	13.33(2.42)	13.09(2.55)	11.35(2.2)	0.001*
QoL(Social)	13.97(2.16)	14.13(2.81)	13.93(3.01)	0.873
QoL(Environment)	13.66(2.25)	13.40(2.82)	11.94(2.25)	0.001*

M, mean; SD, standard deviation; PGSI, Problem Gambling Severity Index; WHOQOL-BREF (HK), Hong Kong Chinese World Health Organization Quality of Life Measure (Abbreviated); QoL, Quality of Life; ^a^Chi-squared test; ^b^One-way ANOVA; ^c^Kruskal-Wallis test; *p<.05.

Significant differences were also found for overall quality of life, physical, psychological and environment QoL (p<0.01 for all). *Post hoc* analysis showed that the high-risk group scored significantly lower than both the low- and moderate-risk groups on overall QoL (p<0.001), physical QoL (p<0.001), psychological QoL (p<0.05), and environment QoL (p<0.05).

### 5.2 Association between gacha gaming behaviors, QoL and problem gambling risk

Correlation analysis revealed a significant positive association between monthly expenses on gacha purchases and total PGSI scores (p<0.05). Conversely, all quality of life domains except for the social domain were significantly and negatively correlated with total PGSI scores (p<0.01). **Table 2** presents the results.

**Table 2:** Correlations between total PGSI score and study variables (N=281).

	Total PGSI score	
	*r*	*p value*
Education level	-0.096	0.110
Monthly income	0.168	0.005
Daily time spent on gacha games	0.094	0.115
Number of gacha games played	0.028	0.637
Frequency of gacha purchases	0.055	0.362
Monthly expenses on gacha purchase	0.128	0.032*
QoL(Overall)	0-.247	0.001*
QoL(Physical)	-0.179	0.003*
QoL(Psychological)	-0.209	0.001*
QoL(Social)	-0.032	0.589
QoL(Environment)	-0.229	0.001*

QoL, Quality of Life; PGSI, Problem Gambling Severity Index; *p<.05.

The regression analysis (**Table 3**) indicated that daily time spent on gacha gaming and social QoL were positively associated with the total PGSI scores (p<0.05). In contrast, overall QoL, education level, and physical QoL were negatively related to the PGSI scores (p<0.05). The final regression model explained 14.2% of the variance in total PGSI scores (Adjusted R²=0.113, F=8.113, p<0.001). The effect sizes (f²) for the significant variables were small, ranging from 0.014 for daily gacha gaming time to 0.04 for social QoL. These findings suggest that gacha gamers with lower education levels, more daily gaming time, and lower overall and physical QoL, combined with higher social QoL, tend to exhibit greater problem gambling tendencies.

**Table 3:** Stepwise multiple regression between gaming variables and quality of life variables for total gambling scores (N=281).

Predictor	Standardized coefficient (*β*)	Unstandardized coefficient (B)	*f* ^2^	t	*p value*
Education level	-.15	-.899	.024	-2.63	.001*
Daily time spent on gacha games	.114	.637	.014	1.98	.048*
QoL(Overall)	-.221	-.970	.032	-3.31	.001*
QoL(Physical)	-.202	-.361	.031	-2.94	.004*
QoL(Social)	.176	.267	.040	2.73	.007*
Model statistics					
R^2^				.129	
Adjusted R^2^				.113	
R				.359	
F				8.113	.001*

QoL, Quality of Life; *p<.05.

## 6 Discussion

### 6.1 Implication of study findings

The current study reveals that overall QoL and its physical domain were negatively associated with problem gambling risk among young adult gacha gamers in Hong Kong. It suggests that gamers with better general and physical wellbeing are less likely to have problem gambling. Conversely, social QoL was positively associated with problem gambling risk, which may indicate that specific peer influences or social dynamics within gaming communities could increase vulnerability. No significant associations were found for psychological and environmental QoL. The mean scores for these QoL domains in the present high-risk problem gambling sample were 11.94-11.35, which were notably lower than those reported for the general adult population in Hong Kong (i.e. 13-14) (37, 38). Their insignificant associations with problem gambling risk suggest that they may be less relevant to gacha gaming’s social and reward-driven dynamics as compared to physical and social QoL.

Additionally, while daily time spent on gacha purchases was a significant predictor, its smallest effect size among all significant predictors in the regression model suggests that it may be a less potent indicator of problem gambling risk as compared to other QoL variables. These findings fill a gap in the existing body of knowledge about the role of quality of life in problem gambling within the context of gacha gaming in Hong Kong. The present findings highlight the importance of paying special attention to gamers with low physical and overall wellbeing, and high social wellbeing to facilitate early identification of at-risk cases in primary healthcare settings.

### 6.2 Social QoL

Our results indicate a positive association between social QoL, as measured by the WHOQOL-BREF, and problem gambling risk in gacha gamers, suggesting that greater social wellbeing may paradoxically encourage compulsive gacha spending and promote gambling behaviors. This finding is consistent with those in the broader gambling literature, such as Oyelade et al. (2023) (39). The mean social QoL scores in the present sample (13.93-14.13 on the 4–20 scale) were comparable to normative data from the Hong Kong general adult population pre- and post-COVID-19 pandemic. Wong et al. (2018) (37) and Census and Population Department (2011) (38) reported a mean social QoL score of 63.96 and 61.44 on a 0–100 scale, respectively, which are equivalent to 13–15 on the 4–20 scale in the general adult population. Hung et al. (2022) (40) reported a mean social QoL of 14.0 on a 4–20 scale among young adults during the COVID-19 pandemic. The consistent findings across different periods may reflect the increasing role of screen-based social interactions, which likely complement traditional forms of social engagement in maintaining perceived wellbeing.

The social QoL reported by gacha gamers, despite their problem gambling risk, can be explained by three mechanisms that challenge traditional views of social wellbeing. First, gacha gaming communities foster a strong group identity where gamers adopt community norms and view themselves as dedicated gamers (41). The acquisition of rare in-game items signals status and commitment, driving increased gaming and spending (42). Second, the emotionally resonant narratives in gacha games can create strong attachments to characters, prompting gacha spending that is driven more by emotional connection than rational decision-making (43). Third, online gaming communities can inadvertently normalize problem gambling behaviors. Gamers sharing outcomes in forums receive praise for successes or sympathy for losses, reinforcing a sense of belonging while embedding in-game spending as routine (42). Features like ‘pity systems’ further integrate in-game spending into the social fabric of the game. These mechanisms suggest that high social QoL scores in this context may reflect engagement with gambling-supportive virtual networks rather than genuine psychological resilience.

The issue of using general QoL instruments to measure gamblers’ wellbeing has been raised by researchers in the gambling field (44). While the WHOQOL-BREF is a well-validated tool for assessing broad domains of QoL, it has notable limitations when applied to behavioral addictions such as Gaming Disorder and problem gambling. As a general measure designed for diverse populations, it lacks sensitivity to the domain-specific harms of these addictions, such as financial pressures, preoccupation with gaming mechanics, and the potentially maladaptive nature of social interactions in virtual environments. Qualitative evidence confirms that gamers often experience anxiety about being “left behind” if they fail to participate in spending cycles, a nuance not captured by standard QoL items (41). In gacha gaming, the general QoL instrument may overestimate social QoL by capturing perceived ‘support’ from online communities that simultaneously normalize and encourage exploitative spending, misinterpreting engagement in such networks as a sign of wellbeing.

This highlights the need to revisit the traditional definition of social QoL in the digital age. While virtual communities can provide a genuine sense of belonging and shared achievement, it is crucial to critically evaluate whether they also embed gambling mechanics that foster dependency and isolation rather than psychological wellbeing. Future research would benefit from developing or adapting gaming-specific QoL instruments capable of distinguishing between resilience-building social connections and engagement in networks that may perpetuate harm.

### 6.3 Overall and physical QoL

The mean physical QoL scores in the present sample were 14.88, 14.76, and 13.13 (on the 4–20 scale) for the low-, moderate-, and high-risk problem gambling groups, respectively. These scores are notably lower than pre-COVID-19 normative data in Hong Kong’s general adult population, with reported mean scores equivalent to 15–16 on a 4–20 scale (37, 38). Furthermore, the mean physical QoL score of the high-risk gambling group (13.13) was also lower than the mean score (13.71) reported among young adults during the COVID-19 pandemic (40). This suggests that gacha gamers, particularly those at high risk for problem gambling, may experience poorer physical wellbeing as compared to the general population.

The negative association between overall and physical QoL and problem gambling risk likely stems from prolonged engagement in gaming activities, which aligns with broader research on other behavioral addictions. A systematic review conducted by Noroozi et al. (2021) identified significant negative correlations between internet addictive behaviors and physical health outcomes, including sleep disturbances and reduced wellbeing (25). Similarly, a meta-analysis by (45) found that gamers with Gaming Disorder often experienced physical health issues such as chronic pain, sleep problems, and decreased physical activity, all of which diminish daily physical wellbeing. In the context of gacha gaming, gamers with poorer physical QoL may turn to gaming as a coping mechanism for negative real-life experiences, such as physical discomfort or fatigue. The immersive nature of gacha games, due to their demanding reward systems, may exacerbate these physical health issues by reducing time for physical activity and rest, further lowering physical QoL and increasing susceptibility to excessive in-game spending.

### 6.4 Daily gaming time

Daily gaming time was found to have a modest yet significant positive association with problem gambling risk in the present study. This finding is consistent with broader research on behavioral addiction, which indicates that prolonged engagement in reward-driven activities can impair cognitive functions related to decision-making. The diminished self-control and irrational spending decisions observed in frequent gacha gamers may stem from impaired inhibitory control caused by prolonged gaming (46). The core mechanic of gacha games, i.e., repeated “pulling” for randomized rewards, likely reinforces this effect by habituating gamers to a cycle of reward-seeking, gradually eroding their resistance to impulsive spending.

### 6.5 Effect sizes for the independent variables

While this study identified an association between certain QoL domains and gacha-related gambling risk, the observed effect sizes were small. Although methodological differences limit direct comparisons with other studies, these modest effect sizes are consistent with findings in the broader literature on behavioral addictions. For instance, a systematic review by Bonfils et al. (2019) noted that QoL variables often yield smaller effect sizes in gambling research, a limitation they attributed to the use of general, non-specific QoL instruments (44).

Furthermore, research consistently shows that protective factors tend to have smaller effect sizes than risk factors. A meta-analysis of Gaming Disorder by Ropovik et al. (2023) found that protective factors, such as life satisfaction, had small effects, whereas risk factors, including anxiety and depression, had moderate effect sizes (47). Similarly, another meta-analysis conducted by Dowling et al. (2017) reported that protective factors for problem gambling, such as parental supervision, had weaker predictive power than risk factors such as poor academic performance and tobacco use (48).

### 6.6 Limitations and recommendations

This cross-sectional study has five significant limitations. First, it employed convenience and snowball sampling, which may introduce selection bias and limit generalizability beyond the predominantly well-educated (78.6% with bachelor’s degrees) Chinese young adults in Hong Kong. Second, the imbalanced sample sizes across the three problem gambling groups, with only 11% (n=31) in the high-risk group, may reduce statistical power and increase the risk of Type II errors, potentially weakening the ability to detect true associations. This imbalance may also explain the small effect sizes of significant independent variables and the low explanatory power of the model. However, protective factors often show smaller effects than risk factors, as explained in Section 6.5. Third, the use of the WHOQOL-BREF, a general quality of life instrument, may not fully capture the unique social dynamics of gacha gaming communities, such as virtual relationships that normalize in-game spending, potentially leading to an overestimation of social QoL. Fourth, the cross-sectional design precludes causal conclusions. Fifth, conducting the study solely in Hong Kong may limit the cultural and contextual applicability of findings to other Asian regions. To address these limitations, future research should adopt longitudinal designs to establish causality, use probability or quota sampling for broader representativeness, include samples from other Asian regions for generalizability, and develop or validate gaming-specific QoL instruments to better capture the social and behavioral nuances of gacha gaming.

## 7 Conclusions

Despite the low explanatory power and small effect sizes in the regression model, this is the first exploratory study looking into the potential association between gacha gaming, quality of life, and problem gambling risk in the context of gacha gaming. This study highlights the need to improve physical health and overall wellbeing to mitigate problem gambling among young adult gacha gamers in Hong Kong. It also points out the importance of addressing social factors, specifically in the gacha gaming community, that reinforce related gambling behaviors. It provides direction for further research and sheds light on the need to evaluate gamers’ quality of life to facilitate the early identification of their problem gambling behavior in primary healthcare settings.

## Data Availability

The raw data supporting the conclusions of this article will be made available by the authors, without undue reservation.

## References

[1] Dang T. The addictive design of mobile gacha games (2023). Available online at: https://urn.fi/URN:NBN:fi:amk-2023081724812 (Accessed May 19, 2025).

[2] Dennis CB Davis TD Chang J McAllister C. Psychological vulnerability and gambling in later life. J Gerontol Soc Work. (2017) 60:471–86. doi: 10.1080/01634372.2017.1329764, PMID: 28494207

[3] 360 research reports. Global gacha games market research report 2023 (2023). Available online at: https://www.360researchreports.com/global-gacha-games-market-27650344 (Accessed May 19, 2025).

[4] Craig C. Global mobile game revenue grew 26% Year-over-year in first nine months of 2020 to nearly $60 billion. Sensor Tower Store Intelligence (2020). Available online at: https://sensortower.com/blog/mobile-game-revenue-and-downloads-q1-q3-2020 (Accessed May 4, 2025).

[5] Montiel I Basterra-González A Machimbarrena JM Ortega-Barón J González-Cabrera J. Loot box engagement: A scoping review of primary studies on prevalence and association with problematic gaming and gambling. PloS One. (2022) 17:e0263177. doi: 10.1371/journal.pone.0263177, PMID: 35085370 PMC8794181

[6] Prasetya DC Proborini E Khanza F Arinda NN. The dynamics of gambling behavior among genshin impact players in Indonesia. (2022). doi: 10.13140/RG.2.2.15857.97121

[7] De Vere K. Japan officially declares lucrative kompu gacha practice illegal in social games (2012). Available online at: https://www.adweek.com/digital/Japan-officially-declares-lucrative-kompu-gacha-practice-illegal-in-social-games/ (Accessed May 19, 2025).

[8] Shibuya A Teramoto M Shoun A Akiyama K. Long-term effects of in-game purchases and event game mechanics on young mobile social game players in Japan. Simul Gaming. (2019) 50:76–92. doi: 10.1177/1046878118819677

[9] Steinmetz F Fiedler I von Meduna M Ante L. Pay-to-win gaming and its interrelation with gambling: Findings from a representative population sample. J Gambl Stud. (2022) 38:785–816. doi: 10.1007/s10899-022-10130-8, PMID: 34106383 PMC9411233

[10] Anselme P Robinson MJF. What motivates gambling behavior? Insight into dopamine’s role. Front Behav Neurosci. (2013) 7:182. doi: 10.3389/fnbeh.2013.00182, PMID: 24348355 PMC3845016

[11] Mills DJ Allen JJ. Self-determination theory, internet gaming disorder, and the mediating role of self-control. Comput Hum Behav. (2020) 105:106209. doi: 10.1016/j.chb.2019.106209

[12] Clark L. Decision-making during gambling: an integration of cognitive and psychobiological approaches. Philos Trans R Soc Lond B Biol Sci. (2010) 365:319–30. doi: 10.1098/rstb.2009.0147, PMID: 20026469 PMC2827449

[13] Vuorinen I Savolainen I Hagfors H Oksanen A. Basic psychological needs in gambling and gaming problems. Addict Behav Rep. (2022) 16:100445. doi: 10.1016/j.abrep.2022.100445, PMID: 35813577 PMC9263400

[14] Drummond A Sauer JD Ferguson CJ Hall LC. The relationship between problem gambling, excessive gaming, psychological distress and spending on loot boxes in Aotearoa New Zealand, Australia, and the United States—A Cross-National Survey. PloS One. (2020) 15:e0230378. doi: 10.1371/journal.pone.0230378, PMID: 32203522 PMC7089530

[15] Zendle D Cairns P. Loot boxes are again linked to problem gambling: Results of a replication study. PloS One. (2019) 14:e0213194. doi: 10.1371/journal.pone.0213194, PMID: 30845155 PMC6405116

[16] Wardle H Zendle D. Loot boxes, gambling and problem gambling among young people: Results from a cross-sectional online survey. Cyberpsychol Behav Soc Netw. (2020) 23:774–9. doi: 10.1089/cyber.2020.0299, PMID: 33103911 PMC8064953

[17] González-Cabrera J Basterra-González A Ortega-Barón J Caba-MaChado V Díaz-López A Pontes HM. Loot box purchases and their relationship with internet gaming disorder and online gambling disorder in adolescents: A prospective study. Comput Hum Behav. (2023) 143:107685. doi: 10.1016/j.chb.2023.107685

[18] Scerri M Anderson A Stavropoulos V Hu E. Need fulfilment and internet gaming disorder: A preliminary integrative model. Addict Behav Rep. (2019) 9:100144. doi: 10.1016/j.abrep.2019.100144, PMID: 31193898 PMC6543453

[19] Raneri PC Montag C Rozgonjuk D Satel J Pontes HM. The role of microtransactions in internet gaming disorder and gambling disorder; A preregistered systematic review. Addict Behav Rep. (2022) 100415. doi: 10.1016/j.abrep.2022.100415, PMID: 35434248 PMC9006671

[20] Tang ACY Lee PH Lam SC Siu SCN Ye CJ Lee RLT. Prediction of problem gambling by demographics, gaming behavior and psychological correlates among gacha gamers: A cross-sectional online survey in Chinese young adults. Front Psychiatry. (2022) 13:940281. doi: 10.3389/fpsyt.2022.940281, PMID: 35990074 PMC9389446

[21] Larrieu M Billieux J Decamps G. Problematic gaming and quality of life in online competitive videogame players: Identification of motivational profiles. Addict Behav. (2022) 133:107363. doi: 10.1016/j.addbeh.2022.107363, PMID: 35689906

[22] Gentile DA Choo H Liau A Sim T Li D Fung D. Pathological video game use among youths: a two-year longitudinal study. Pediatrics. (2011) 127:e319–29. doi: 10.1542/peds.2010-1353, PMID: 21242221

[23] Rehbein F Baier D. Family-, media-, and school-related risk factors of video game addiction. J Media Psychol. (2013) 25:118–28. doi: 10.1027/1864-1105/a000093

[24] World Health Organization. The World Health Organization quality of life (WHOQOL) (2025). Available online at: https://www.who.int/publications/i/item/WHO-HIS-HSI-Rev.2012.03 (Accessed May 19, 2025).

[25] Noroozi F Hassanipour S Eftekharian F Eisapareh K Kaveh MH. Internet addiction effect on quality of life: A systematic review and meta-analysis. Sci World J. (2021) 2021:2556679. doi: 10.1155/2021/2556679, PMID: 34912181 PMC8668296

[26] Jeong YW Han YR Kim SK Jeong HS. The frequency of impairments in everyday activities due to the overuse of the internet, gaming, or smartphone, and its relationship to health-related quality of life in Korea. BMC Public Health. (2020) 20:1–16. doi: 10.1186/s12889-020-08922-z, PMID: 32552690 PMC7301989

[27] Kwok C Leung PY Poon KY Fung XC. The effects of internet gaming and social media use on physical activity, sleep, quality of life, and academic performance among university students in Hong Kong: A preliminary study. Asian J Soc Health Behav. (2021) 4:36–44. doi: 10.4103/shb.shb_81_20

[28] Loo JM Shi Y Pu X. Gambling, drinking and quality of life: Evidence from Macao and Australia. J Gambling Stud. (2016) 32:391–407. doi: 10.1007/s10899-015-9569-3, PMID: 26337063

[29] de Oliveira Pinheiro B Monezi Andrade AL Lopes FM Reichert RA de Oliveira WA da Silva AM. Association between quality of life and risk behaviors in Brazilian adolescents: an exploratory study. J Health Psychol. (2022) 27:341–51. doi: 10.1177/1359105320953366, PMID: 32878479

[30] Ferrari Junior GJ Silva ABD Meneghetti A Leite CR Brust C Moreira GC. Relationships between internet addiction, quality of life and sleep problems: a structural equation modeling analysis. J Pediatr. (2024) 100:283–8. doi: 10.1016/j.jped.2023.11.009, PMID: 38182125 PMC11065653

[31] Paulus FW Ohmann S Von Gontard A Popow C. Internet gaming disorder in children and adolescents: a systematic review. Dev Med Child Neurol. (2018) 60:645–59. doi: 10.1111/dmcn.13754, PMID: 29633243

[32] STROBE. STROBE: Strengthening the reporting of observational studies in epidemiology – What is STROBE? (2025). Available online at: https://www.strobe-statement.org/.

[33] Cohen J. A power primer. Psychol Bull. (1992) 112:155–9. Available online at: https://psycnet.apa.org/doi/10.1037/14805-018 (Accessed June 3, 2025). 10.1037//0033-2909.112.1.155 19565683

[34] Loo JM Oei TP Raylu N. Psychometric evaluation of the problem gambling severity index-Chinese version (PGSI-C). J Gambl Stud. (2011) 27:453–66. doi: 10.1007/s10899-010-9221-1, PMID: 20924655

[35] Leung KF Tay M Cheng S Lin F. Hong Kong Chinese version World Health Organisation Quality of Life – Abbreviated version. Hong Kong SAR: Hong Kong Hospital Authority (1997).

[36] Leung KF Wong WW Tay MSM Chu MML Ng SSW. Development and validation of the interview version of the Hong Kong Chinese WHOQOL-BREF. Qual Life Res. (2005) 14:1413–9. doi: 10.1007/s11136-004-4772-1, PMID: 16047516

[37] Wong FY Yang L Yuen JW Chang KK Wong FK. Assessing quality of life using WHOQOL-BREF: a cross-sectional study on the association between quality of life and neighborhood environmental satisfaction, and the mediating effect of health-related behaviors. BMC Public Health. (2018) 18:1113. doi: 10.1186/s12889-018-6007-8, PMID: 30208869 PMC6134517

[38] Census and Population Department, HKSAR. 2011 population census, district profiles. Hong kong: Census and Population Department (2011). Available online at: http://www.census2011.gov.hk/en/constituency-area.html (Accessed June 3, 2025).

[39] Oyelade OO Ojuolape MO Naseem AR Fehintola VA. Impact of gambling on the mental and social wellbeing of youths in Ibadan, Nigeria. Afr J Humanities Contemp Educ Res. (2023) 13:1–11. Available online at: https://publications.afropolitanjournals.com/index.php/ajhcer/article/view/655 (Accessed June 3, 2025).

[40] Hung MSY Ng WWM Choi EKY. The impacts of the COVID-19 pandemic on Hong Kong nursing students’ mental health and quality of life. Int J Environ Res Public Health. (2022) 19:15117. doi: 10.3390/ijerph192215117, PMID: 36429837 PMC9690710

[41] Hong TY. They are just 2D pixels” Understanding the Motivation behind Gacha Gamers. Nanyang Technological University, Singapore (2023).

[42] Sirola A Savela N Savolainen I Kaakinen M Oksanen A. The role of virtual communities in gambling and gaming behaviors: A systematic review. J Gambl Stud. (2021) 37:165–87. doi: 10.1007/s10899-020-09946-1, PMID: 32306232 PMC7882555

[43] Johnson MR Brock T. The ‘gambling turn’ in digital game monetization. J Gaming Virtual Worlds. (2020) 12:145–63. doi: 10.1386/jgvw_00011_1

[44] Bonfils NA Aubin HJ Benyamina A Limosin F Luquiens A. Quality of life instruments used in problem gambling studies: A systematic review and a meta-analysis. Neurosci Biobehav Rev. (2019) 104:58–72. doi: 10.1016/j.neubiorev.2019.06.040, PMID: 31271803

[45] Männikkö N Ruotsalainen H Miettunen J Pontes HM Kääriäinen M. Problematic gaming behaviour and health-related outcomes: A systematic review and meta-analysis. J Health Psychol. (2020) 25:67–81. doi: 10.1177/1359105317740414, PMID: 29192524

[46] Kowalik BA Delfabbro P. A scoping review of impaired control in the context of gaming disorder: Conceptualisations across different studies and frameworks. Int J Ment Health Addict. (2025), 1–36. doi: 10.1007/s11469-025-01454-w

[47] Ropovik I Martončik M Babinčák P Baník G Vargová L Adamkovič M. Risk and protective factors for (internet) gaming disorder: A meta-analysis of pre-COVID studies. Addict Behav. (2023) 139:107590. doi: 10.1016/j.addbeh.2022.107590, PMID: 36571943

[48] Dowling NA Merkouris SS Greenwood CJ Oldenhof E Toumbourou JW Youssef GJ. Early risk and protective factors for problem gambling: A systematic review and meta-analysis of longitudinal studies. Clin Psychol Rev. (2017) 51:109–24. doi: 10.1016/j.cpr.2016.10.008, PMID: 27855334

